# Supplementary material for: Coexistence of state, choice, and sensory integration coding in barrel cortex LII/III
Source: Nat Commun. 2024 Jun 5;15:4782. doi: 10.1038/s41467-024-49129-9 (PMC11153558; doi:10.1038/s41467-024-49129-9)
Supplement: Supplementary file 1 — Supplementary Information [file 41467_2024_49129_MOESM1_ESM.pdf]

Supplementary Information for

Coexistence of state, choice, and sensory integration coding in barrel cortex LII/III

Pierre-Marie Gardères*, Sébastien Le Gal, Charly Rousseau, Alexandre Mamane, Dan Alin Ganea, Florent Haiss*

* Correspondence pmgarderes@gmail.com (P.M.G.), florent.haiss@pasteur.fr (F.H.).

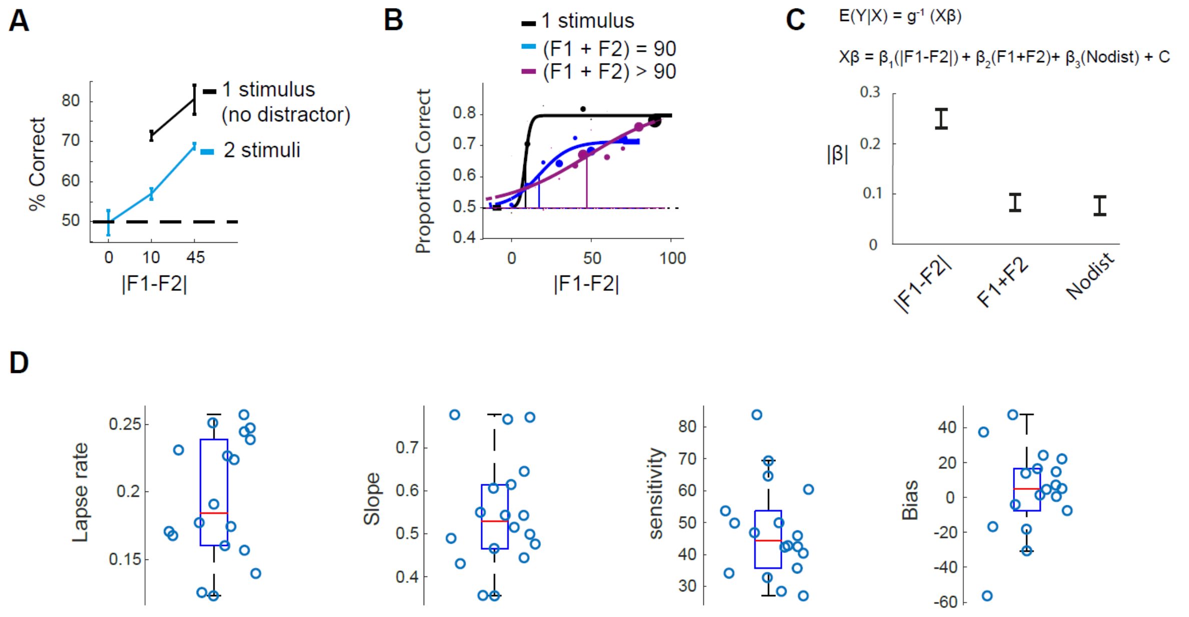

Figure S1. Behavioral performance in the stimulus space

A, Average performance across trials with single (black) or dual (blue) frequency stimulation. In details: at ΔF =0, F1/F2 is equal to 45/45 (blue). At ΔF =10, F1/F2 is equal to 50/40(blue) or 10/0 (black). At ΔF =45, F1/F2 is equal to 45/0 (black) or 90/45 (blue).

B, Psychometric fit of the proportion correct P(correct) for different categories of stimuli. Vertical bar represents sensitivity at half of the fitted "lapse rate". Data point size represents the relative amount of trials (normalized within each 3 conditions). Discrimination seems easiest when a single stimulus is presented (black). When two stimuli are presented on W1 and W2 simultaneously, discrimination seems harder when the sum of the two frequency is high (F1 + F2 >90Hz, magenta versus blue).

C, The F1-F2 difference is the most important stimulus parameter for behavioral categorization. Influence of the stimulation parameters on the subjects' performance (average across pooled trials from all experiments, n=166599 trials). The response boolean vector Y (success =1, failure =0) is fitted with a generalized linear model, assuming a binomial distribution and using a logit link function. All three regression variables were Z-scored so that their ß weight are comparable. Note that we compare absolute ß weight. C is a constant. Error bars represent a 95% confidence interval.

D, Parameters of the psychometric fit for 18 animals; number of trials per animal: average 6370.5; ranging from 1958 to 13260.

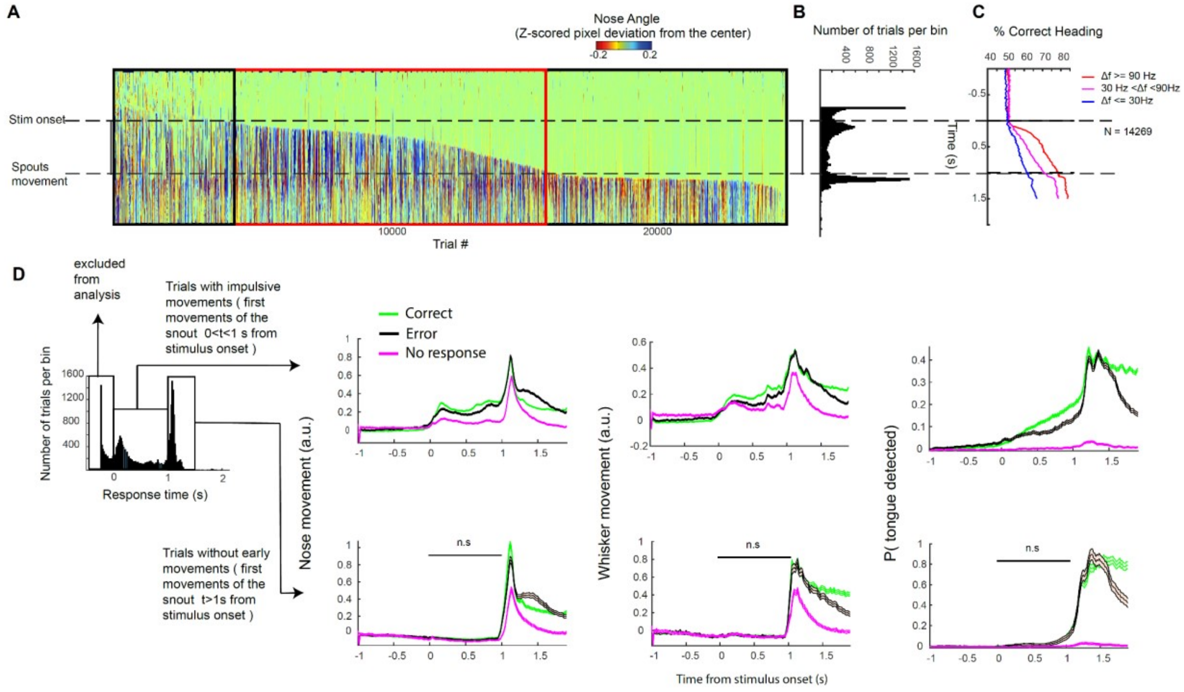

Figure S2, Reaction times and facial movements

A, Nose heading direction as a function of time. Trial # in x, time in y, blue indicates left heading nose; red indicates right heading nose; trials sorted according to the first detected nose movement (see methods).
B, Distribution over time of first detected nose movement.

C, Heading direction predicts the correct target side for easy (red), middle (magenta) and difficult trials (blue).

D, Body movement extracted from video analysis (see methods). Left: trial sorting according to nose reaction time. Right, top row: body movement during impulsive trials. Right, bottom row: body movements during late response trials. p-value indicates difference of movement between the three behavioral categories (Friedman test, n = 7 animals). Error shades represent a 95% confidence interval following a normal distribution on a trial-by-trial basis (n= 30300 trials from 7 animals).

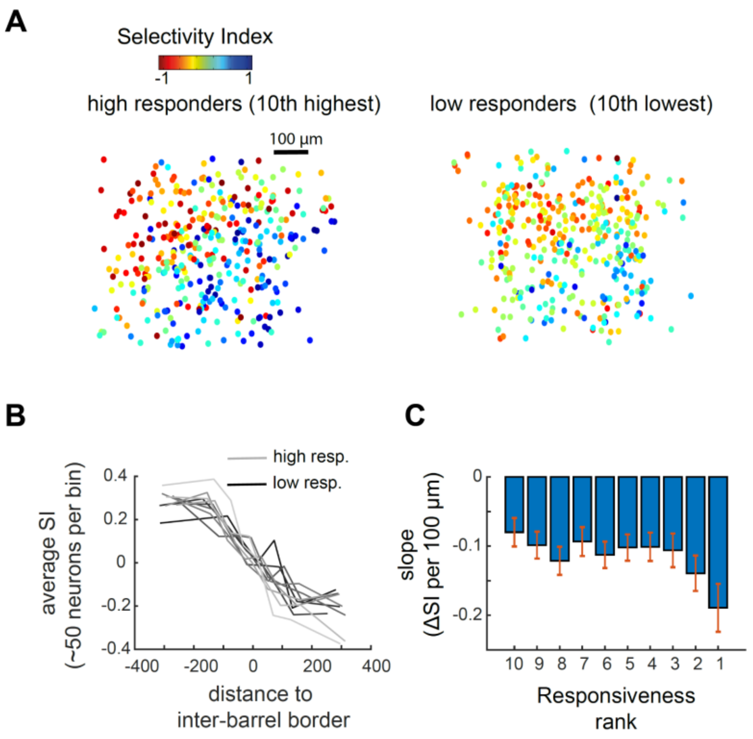

Figure S3. Highly responsive neurons are more whisker selective and clustered toward the barrel center.

A, Spatial distribution of the selectivity from highest and lowest responding neurons (left and right respectively).

B, SI as a function of distance to the two-barrel separation depends on responsiveness of the neurons. From lightest to darkest color line represents neurons split in ten deciles going from the most to least responsive neurons. Data is further split in bins of ~50 microns width and finally averaged to allow plotting the spatial dependence of SI.

C, Slopes from a linear regression of the data in B, between distance to barrel and selectivity. Selectivity of highly responsive cells (Responsiveness rank 1) changes more rapidly with distance to the barrel center. Error bars represent CI95.

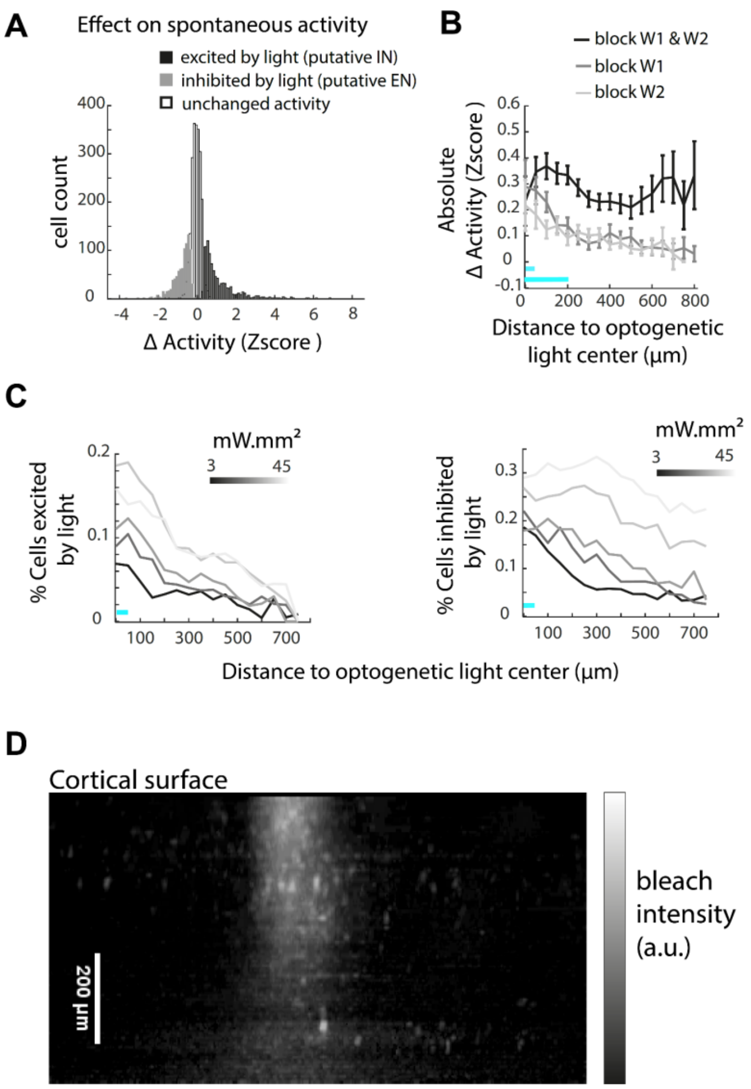

Figure S4. Spatial spread of optogenetic excitation/ inhibition

A, Change in fluorescence in response to optogenetic light (450 µm disk) for all cells in the FOV (n= 5034 neurons, 3 animals, 5 FOV). 23.2% of cells showed activation by optogenetic light (putative inhibitory neurons) and 23.8% cells showed inhibition (putative excitatory neurons) compared to spontaneous activity.

B, Change in activity as a function of distance to center of inhibition, quantified as absolute change in activity |ΔZscore|, for the three optogenetic conditions with the smallest light amplitude (e = 1.4 mW.mm^2 and e= 3 mW.mm^2 for the 450 µm and 105 µm disks respectively).

C, Inhibition and excitation as a function of distance to illumination center during selective barrel illumination.

D, Visualization of the optogenetic light spread in the axial dimension of the microscope. The light spread is measured as bleaching induced on GCaMP6s expressed across cortical layers. Bleaching was induced by prolonged exposure to the optogenetic light pattern (12 minutes of continuous illumination, 105 µm disk FWHM, at ~ 44 mW/mm^2). Pixel intensity is computed as fluorescence before exposition minus fluorescence after exposition, thus bleaching appearing as a brighter column. Fluorescence was measured before and after bleaching with the same two photon imaging parameters in a stack with 5µm steps. Each plane is averaged over 1 second. Volume rotation and visualization was performed with ImageJ.

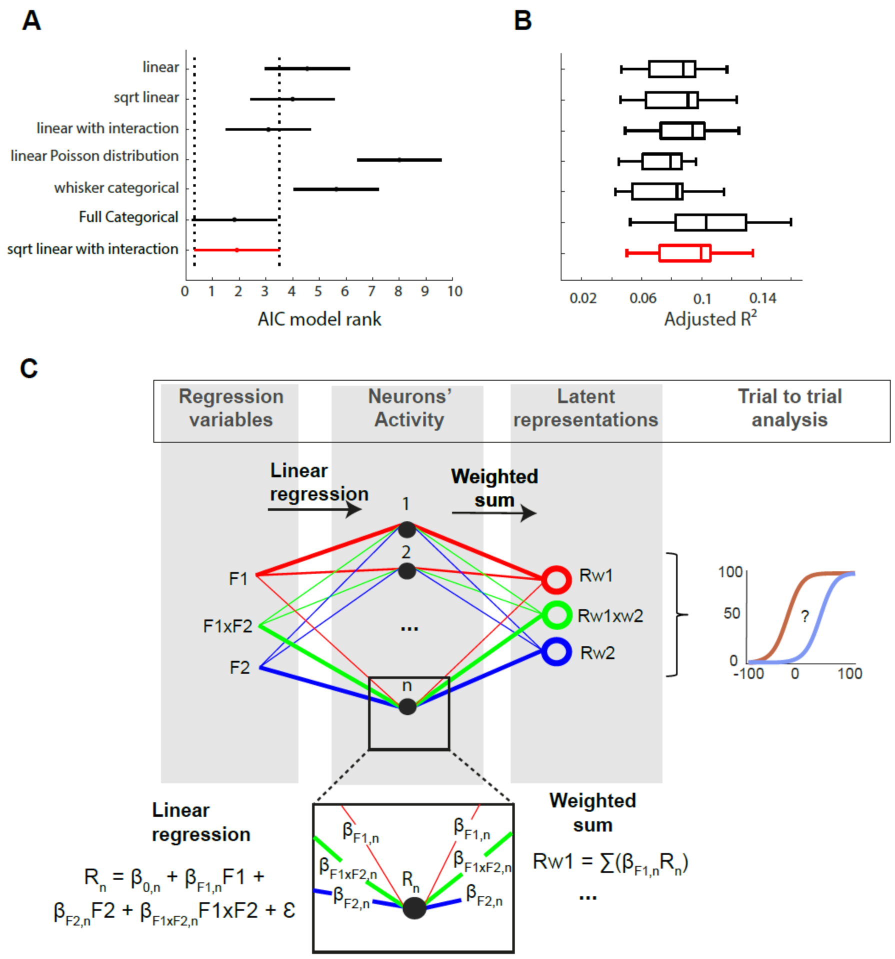

Figure S5. The square root linear model with interaction yields similar results to the full categorical model for predicting single neuron's activity.

A, Comparison of Akaike criterion (fit performance given complexity of the model). Friedman's test with multiple comparison, shows that no other model tested outperform the model used in the rest of the study (sqrt linear with interaction; described in Equation (1); see methods).
B, Variance explained for different linear, categorical, and non-linear models of activity. The higher variance explained by the full categorical model could indicate that some neurons have a modal tuning to specific frequencies.
C, Graphical summary of population analysis used in Fig 4-7.
In the initial step, linear regression is independently applied to each neuron (Rn representing the activity of neuron n) to ascertain the sensory features it represents. This regression process identifies the weights ß$_{F1,n}$, ß$_{F2,n}$, ß$_{F1xF2,n}$, which each of the three sensory features (F1, F2 and F1xF2) drive the activity of neuron n. ß$_{0,n}$ represents the baseline activity in the model, and Ɛ represents the non-fitted residuals. In the subsequent step, the activities of multiple neurons are pooled using the same sensory weights that drive them. These weighted pools embody the latent representations of stimulus features encoded by the populations of neurons: Rw1 represents the population response to the stimulation frequency of whisker 1 (F1), Rw2 corresponds to the population response to the stimulation frequency of whisker 2 (F2), and Rw1xw2 carries the population response to the cross-whisker interaction. These latent representations are then leveraged for various analyses, including decoding, exemplified here as neurometric analysis, or for the trial-by-trial representational geometry of sensory versus choice signals, as depicted in Figures 6 and 7. Additional regression variables, namely choices (C1 and C2) or engagement (Eng), are incorporated explicitly in the models presented in Figure 6 and 7 respectively, to generate additional latent representations of choices and engagement (not represented here, described in the methods).

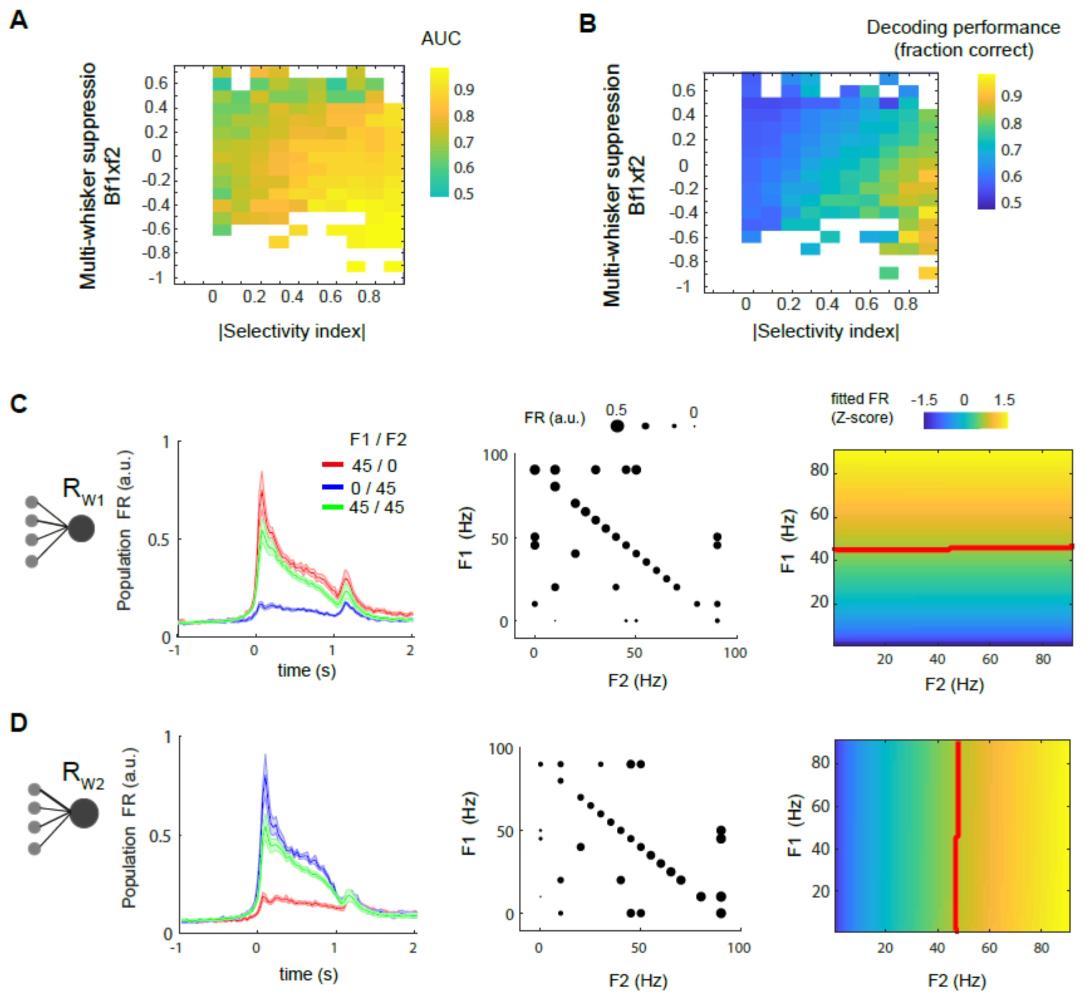

Figure S6: **Multi-whisker (MW) suppression improves decoding performance at the single neuron level, and has little net effect on the whole population average.**

A, Discrimination of F1>F2 over the stimulus space depends on selectivity and MW suppression across neurons. Discrimination is measured as AUC of F1>F2 over the entire stimulus space, as described for Fig 4E. The pixel colors represent the average AUC value for neurons in the bin. The neuronal population from all animals is split into bins defined in two dimensions on their MW suppression (BF1xF2; y-axis) and their absolute selectivity index (SI; x-axis). Note the increase of AUC along both x and y axis, suggesting the MW suppression improves discriminability independent of its relationship with whisker selectivity.

B, Decoding performance depends on selectivity and MW suppression. Decoding performance is quantified as the fraction of correctly decoded trials. Decoding was performed with a GLM using logit as the link function and assuming binomial distribution of F1>F2. Binning performed as in A. Please note the increase of AUC along both the x and y axis.

C-D, Neural population pools Rw1 and Rw2 respond mostly to whisker 1 and 2 but also slightly to the adjacent whisker. Rw1 and Rw2 show sublinear integration of the two whiskers stimulated simultaneously. Rw1 and Rw2 were computed from two independent pools of neurons based on the sign of Bf1 -Bf2. Error shades represent s.e.m. across FOV. Firing rate and fitted firing rate in the middle and right plots are averaged across FOV. Note that the model with interaction does not fully account for the population firing rate which might be due to the diverse tuning within the population, including MW enhancement (Fig. 4b).

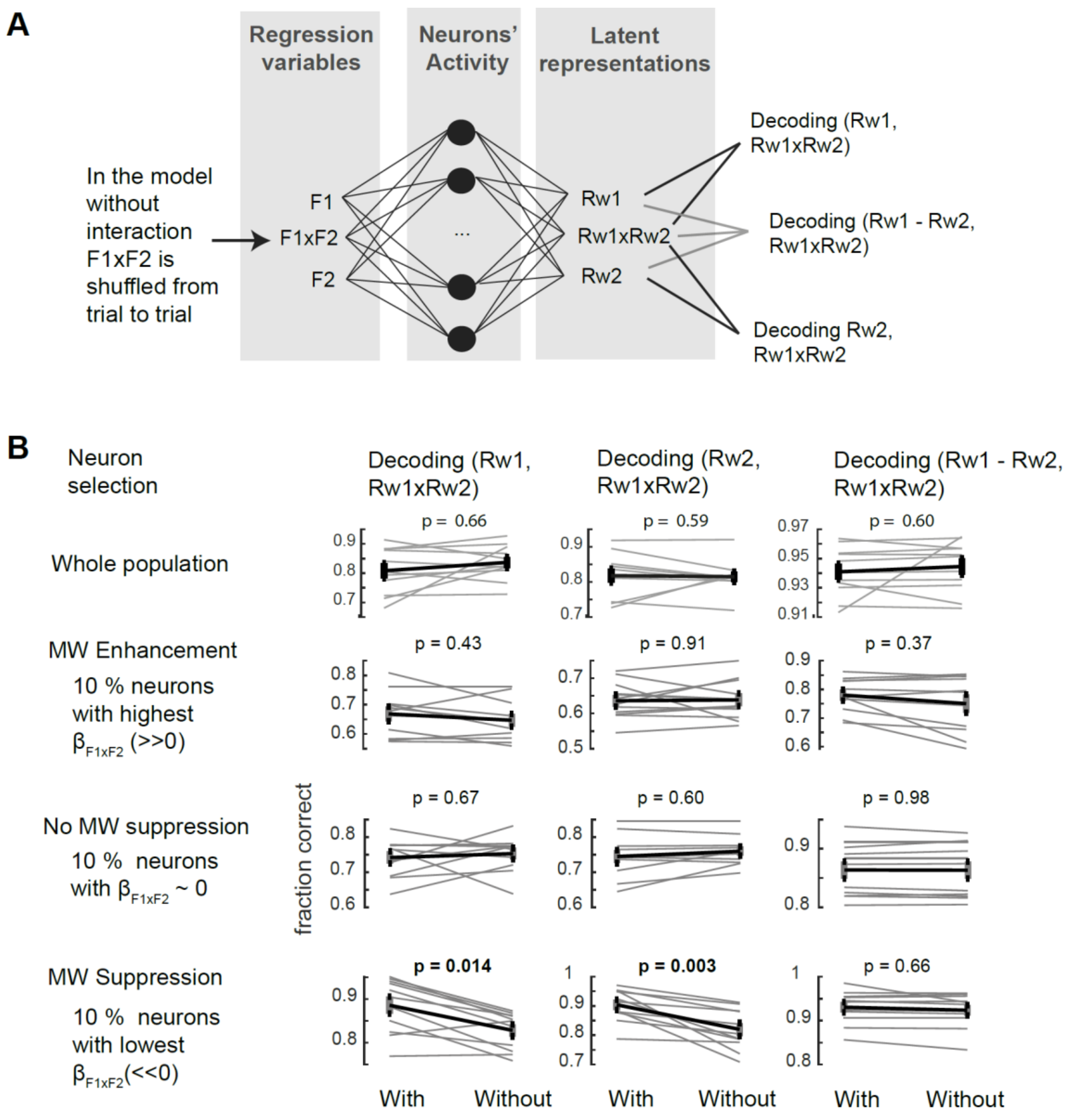

Figure S7: Impact of multi-whisker (MW) interaction term on (sub)-population decoding performance.

A, Schematic procedure for decoding **with** or **without** interaction. When decoding without interaction, the regression variable F1xF2 is shuffled from trial to trial in the model without interaction. Each neurons activity is fitted trial per trial as described in Fig. 4. Activity of all neurons (or subpopulations) is combined into Rw1, Rw2 and Rw1xw2, (respectively latent representations of F1, F2 and F1xF2). The target side (defined by the sign of F1-F2) is decoded separately by three combinations of latent variables. Decoding is performed using a GLM with logistic regression.

B, Comparison of decoding **with** or **without** interactions in different subpopulations of neurons (rows) with decoding of different combinations of latent variables (columns). Subpopulations contain 10 % of neurons with most MW enhancement (top), 10 % null MW interaction (middle) or 10 % most MW suppression (bottom). The interaction term significantly improves decoding performance for the subpopulation of neurons with MW suppression. P-value is computed with a linear mixed effect model across FOV using mice identity as grouping variable.

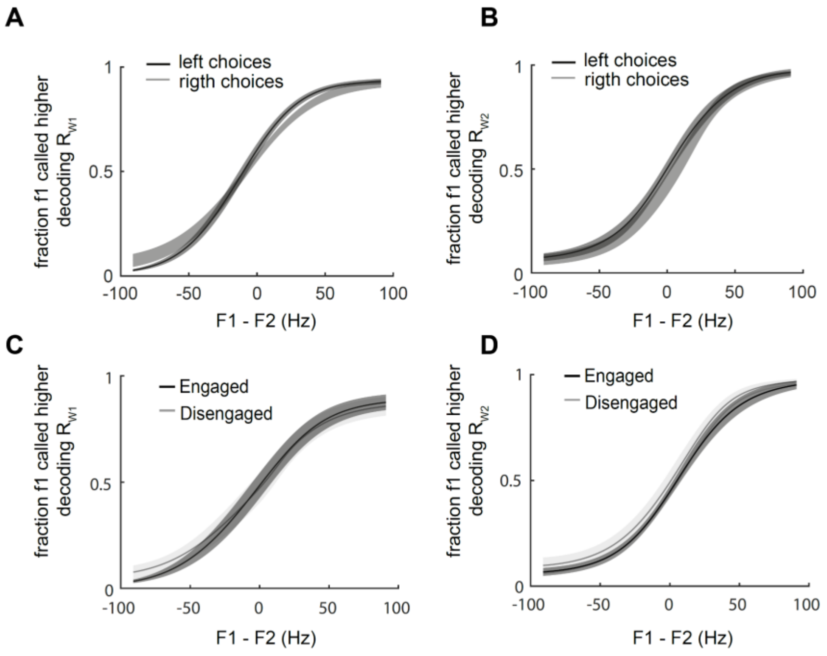

Figure S8: **Neurometric functions of Rw1 and Rw2**

A, B, Neurometric functions in trials with left versus right response from the animal. Decoding of Rw1 (A) or RW2 (B).

C, D, Neurometric functions in trials with or without responses. Comparison of slope and bias compared with a Wilcoxon sign-rank test reveals no significant difference ($p>0.05$, $n = 11$ FOVs). Decoding of Rw1 (C) or RW2 (D).

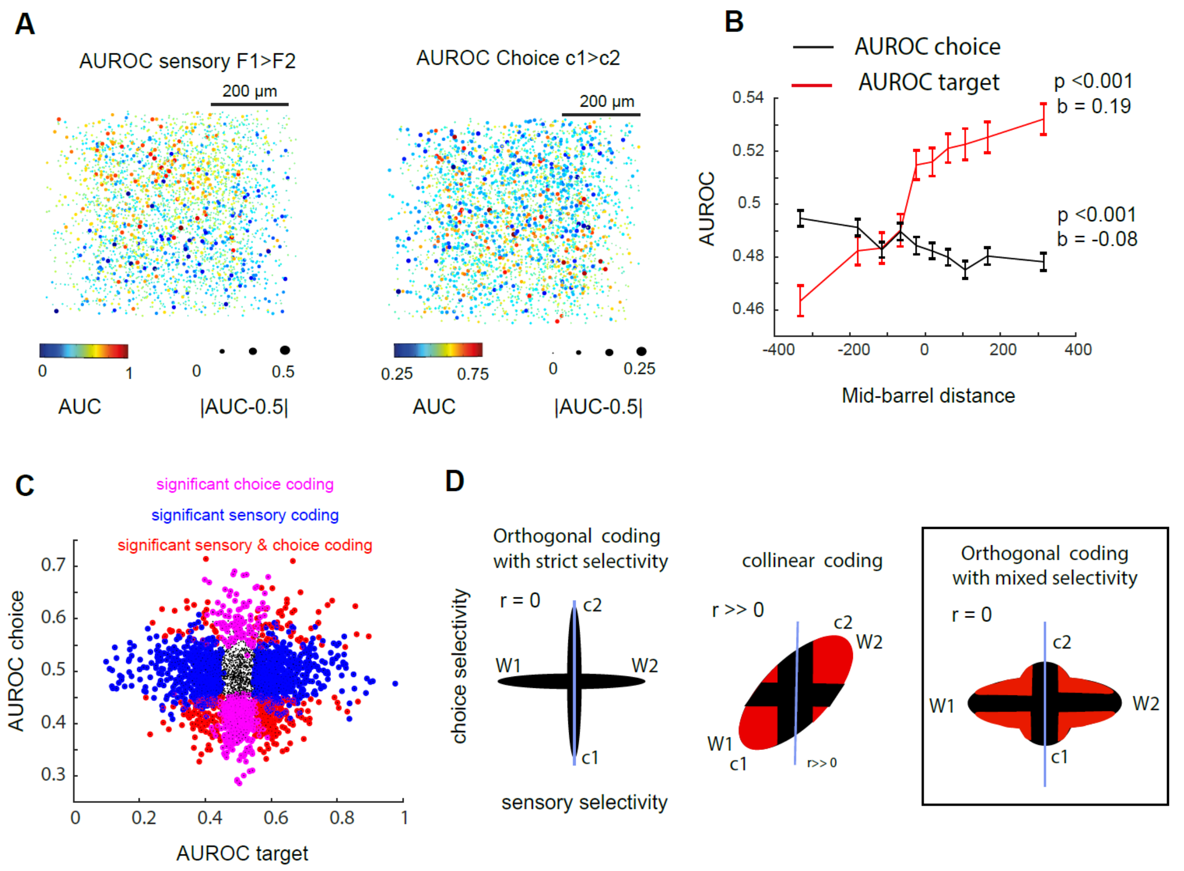

Figure S9: Somatotopy of choice; mixed and orthogonal sensory/choice coding

A, All neurons from 11 FOVs aligned to the mid-points between barrels. Left: AUROC for sensory discrimination (F1>F2 versus F1<F2); Right: AUROC for choice discrimination (C1 versus C2). Size of the dot represent deviation from chance level AUROC (0.5); color represent preference for whisker 1 (>0.5) or whisker 2 (<0.5). Note that size and color are normalized differently on the left and right plots with AUROC target having stronger deviation from chance.

B, Reversed somatotopy for AUROC choice as compared to AUROC target. A LME model is used to test dependence of AUROC on position along the inter-barrel-axis (i.e. the diagonal between W1 and W2 barrel center). For graphical display, we split all neurons in ten bins of equal size. P values (p) and estimates of effect size (b) are computed with a LME model, using n = 3118 neurons from 5 animals (grouping variable).

C, AUROC choice as a function of AUROC target. Statistical significance is tested independently for each neuron as being <0.025 or >0.025 from a distribution of AUROC with shuffled trial identity. As described in the text, we find a bias with more neurons preferring choice 1 (AUROC <0.5). This illustrate that most neurons code for either choice (magenta) or sensory target (blue), with a fraction of neurons having significant sensory and choice selectivity (red).

D, Three scheme of possible sensory/choice coding across the neuronal population. Red areas represent significant selectivity for both sensory and choice variables. Our data indicates that coding of the two variables is orthogonal with mixed selectivity.

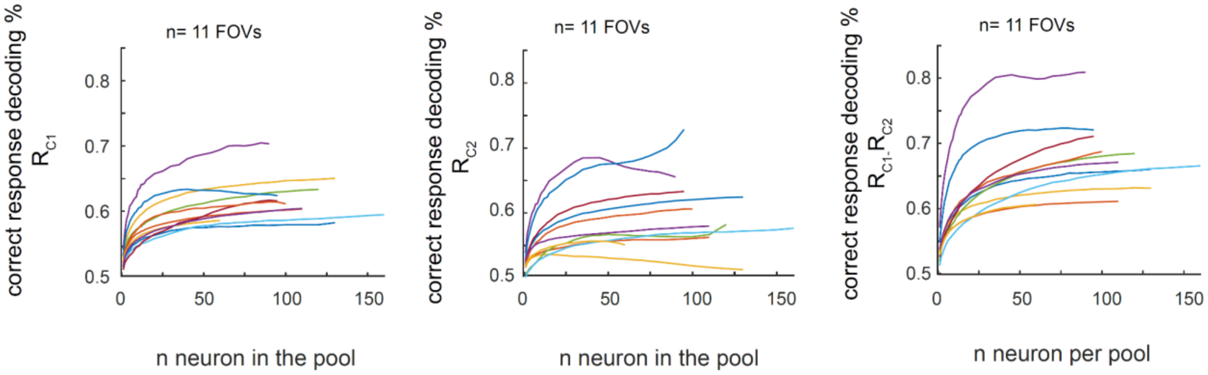

Figure S10 choice information in sub-populations R_{C1}, R_{C2} and R_{C1} - R_{C2}.

Performance of choice discriminability as a function of the number of neurons included in the pooled response. From left to right: discriminability of R_{C1}, $R_{C2,}$ and R_{C1} - R_{C2}. Each line represents data from one FOV (n = 11 FOV). Matched number of Choice 1/Choice 2 trials in each stimulus conditions. Decoding is 10-fold cross-validated.

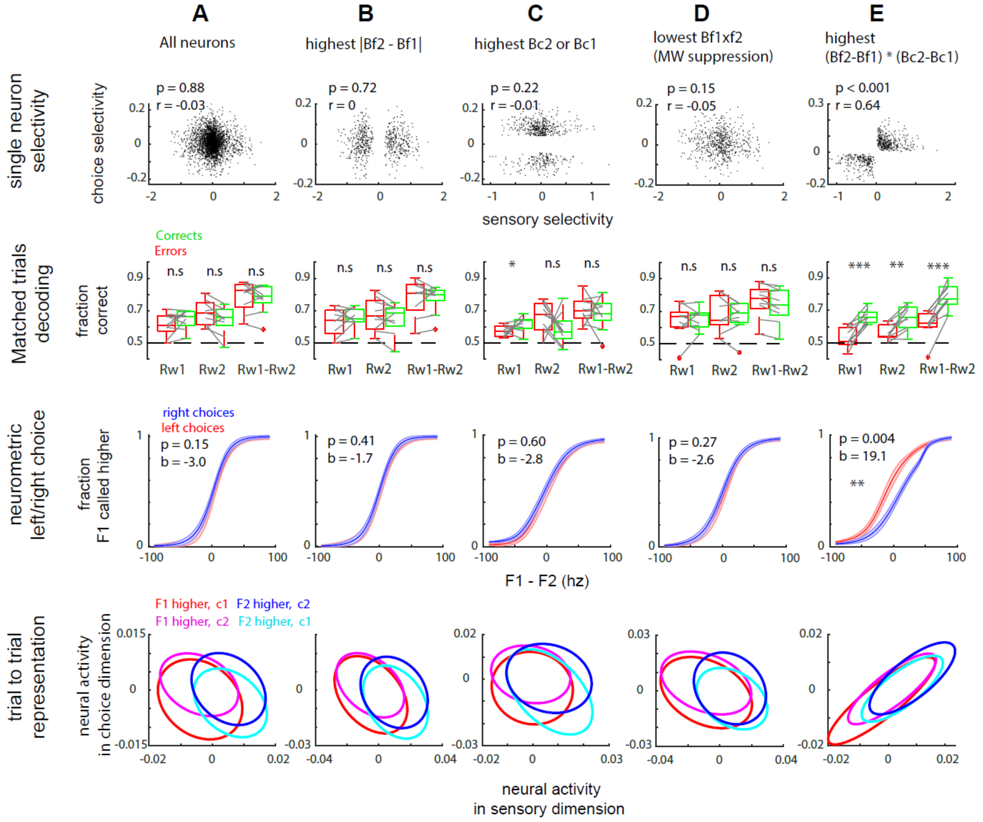

Figure S11: Analysis of sensory choice coding in sub-populations of neurons with strong selectivity.

We compared sensory/choice coding of the entire population (A) to 4 subpopulations that code best for sensory selectivity (B), choice selectivity (C), multi-whisker suppression (D), or intersection of sensory features and choice in correct trials (E). For the (E) population only, sensory and choice become non-orthogonal. Selection criteria are indicated on top of the column. 20% with highest criterion were included in analysis (B) to (E). (E) is exemplary of collinear coding of sensory and choice variable that leads to better decoding of the target in correct trials.

From top row to bottom: (row 1) Single neurons' selectivity for choice as a function of selectivity for whisker, quantified as $ß_{C1}$-$ß_{C2}$ and $ß_{F1}$ -$ß_{F2}$ respectively. (row 2) Sensory decoding performance (F1>F2) from Rw1, Rw2 and Rw1-Rw2 compared between correct versus error trials in matching stimulus condition (with $|\Delta F|<30$). LME model analysis * indicates $p<0.05$; ** indicates $p<0.01$ and *** indicates $p<0.001$. (row 3) neurometric performance in left and right choice trials. (row 4) trial to trial representation of different trial categories (color coded) spanning the four possible combinations of target and choice side. Note the collinearity of representation and the representational angle between choices being ~45 degree for (E) only.

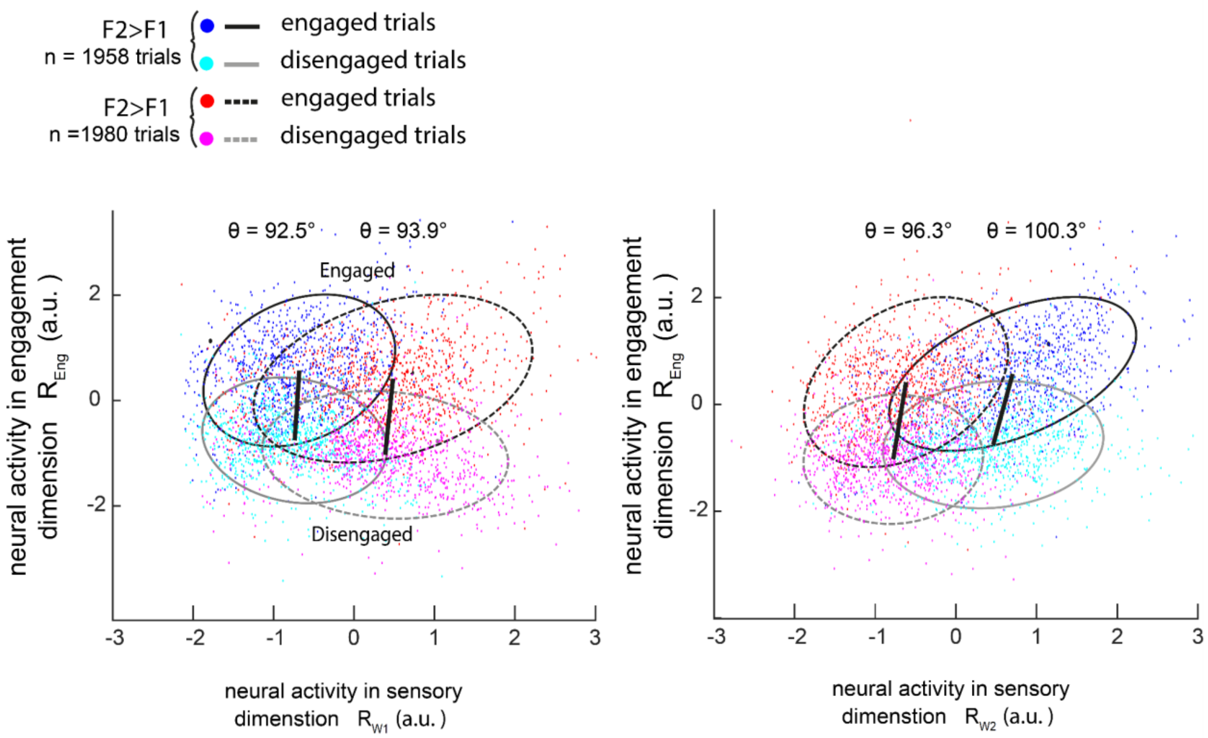

Figure S12. Engagement changes the sensory gain in Rw1 and Rw2 similarly in trials F1>f2 and F2>F1

Neural activity during engaged and disengaged states, in the sensory and engagement dimensions. Left: engagement versus W1 neural representation (Rw1). Right: engagement versus W2 neural representation (Rw2). Same method and description as in Fig. 7e. but both F1>F2 and F2>F1 trials are included in the same panel. Bars represent transition from engaged and disengaged trials. Note that all representational angles are tilted to the right, showing an increase in response of Rw1 and Rw2 during engagement across stimulation conditions (i.e. independent of the whisker stimulated at the highest frequency).

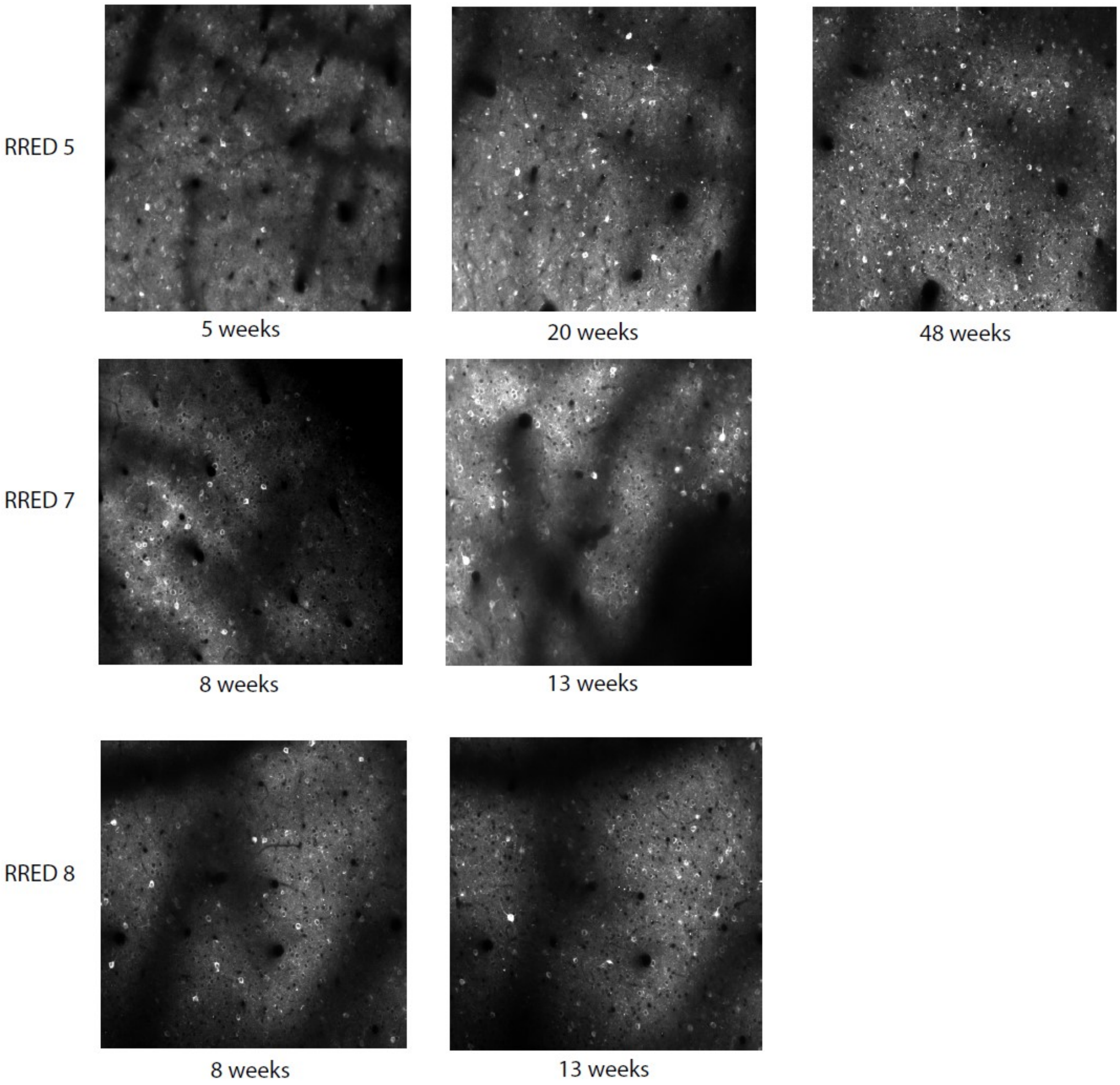

Figure S13. Example FOVs showing healthy jRGECO expression between 5 and 48 weeks following injections.

Animals RRDED 1, RRED2 and RRED8, different Fields of view. Most of our imaging was carried out before 14 weeks (with the exception of 1 FOV). Note the apparition of fluorescent aggregate in the 48 weeks example.

Field of view/ Dataset	Animal/ stimulated whiskers	depth of recording / days of imaging	virus/ indicator	numbers of neurons (type)	FOV size / pixels / imaging rate
sp1 /2pbehavior	RRED1 / C1-D1	~150µm / 56-75 (1 day /2)	jRGECO1a	342 (syn[1])	ML[2]: 738; AP: 605 / 30Hz
sp2 /2pbehavior	RRED1 / C1-D1	~180µm / 59-76 (1 day /2)	jRGECO1a	299 (syn)	ML: 590; AP: 484/ 30Hz
sp3 /2pbehavior	RRED2 / C1-D1	~125µm / 58-76 (1 day /2)	jRGECO1a	470 (syn)	ML: 738; AP: 605 / 30Hz
sp4 /2pbehavior	RRED2 / C1-D1	~160µm / 60-77 (1 day /2)	jRGECO1a	282 (syn)	ML: 590; AP: 484/ 30Hz
sp5 /2pbehavior	RRED5 / C1-D1	~120µm / 52-72 (1 day /2)	jRGECO1a	390 (syn)	ML: 738; AP: 605 / 30Hz
sp6 /2pbehavior	RRED5 / C1-D1	~150µm / 55-72 (1 day /2)	jRGECO1a	289 (syn)	ML: 590; AP: 484/ 30Hz
sp7 /2pbehavior	RRED5 / C1-D1	~180µm / 113-130 (1 day /2)	jRGECO1a	291 (syn)	ML: 738; AP: 605 / 30Hz
sp8 /2pbehavior	RRED6 / B1-C1	~140µm / 58-61	jRGECO1a	328 (syn)	ML: 738; AP: 605 / 30Hz
sp9 /2pbehavior	RRED7 / B1-C1	~130µm / 58-89 (1 day /2)	jRGECO1a	371 (syn)	ML: 738; AP: 605 / 30Hz
sp10 /2pbehavior	RRED8 / B1-C1	~130µm / 57-88 (1 day /2)	jRGECO1a	385 (syn)	ML: 738; AP: 605 / 30Hz
sp11 /2pbehavior	GADRED1 / C1-D1	~130µm / 74-88 (4 days total)	jRGECO1a; EGFP	259 (syn)	ML: 738; AP: 605 / 30Hz
sp1 /2p Inhib	GADRED1 / C1-D1	~100-140µm -180 / 20 -25	jRGECO1a; EGFP	300 (syn) of which 49 (GAD+) 183 (GAD-)	ML: 590; AP: 484/ 10Hz (3 planes)
sp2 /2p Inhib	GADRED1 / C1-D1	~120-160µm -200 / 21 -26	jRGECO1a; EGFP	359 (syn) of which 58 (GAD+) 235 (GAD-)	ML: 590; AP: 484/ 10Hz (3 planes)
sp3 /2p Inhib	GADRED1 / C1-D1	~130µm / 74-88 (4 days total)	jRGECO1a; EGFP	258 (syn) of which 57(GAD+) 138(GAD-)	ML: 738; AP: 605 / 30Hz
sp4 /2p Inhib	GADRED2 / C1-D1	~100-140µm -180 / 23 -27	jRGECO1a; EGFP	211(syn) of which 14(GAD+) 258(GAD-)	ML: 590; AP: 484/ 10Hz (3 planes)
sp5 /2p Inhib	GADRED2 / C1-D1	~120-160µm -200 / 23 -27	jRGECO1a; EGFP	194 (syn) of which 16 (GAD+) 139 (GAD-)	ML: 590; AP: 484/ 10Hz (3 planes)
sp6 /2p Inhib	VGATEGFP13 / B1-C1	~120µm / 21	jRGECO1a; EGFP	248 (syn) of which 73(VGAT+) 121(VGAT-)	ML: 590; AP: 484/ 10Hz (3 planes)
sp1/Opto2p (dual)	VGATCHRCAMP1 / C1-D1	~110-130-150-170-190µm / 121-123	GCaMP6s hChr2-EYFP	2448 (syn)	ML: 948; AP: 784/ 4.6 Hz (5 planes)
sp2/Opto2p (selective)	VGATCHRCAMP1/C1-D1	~110-130-150-170-190µm / 124-132	GCaMP6s hChr2-EYFP	1592 (syn)	ML: 948; AP: 784/ 4.6 Hz (5 planes)
sp3/Opto2p (dual)	VGATCHRCAMP2/B1-C1	~110-130-150-170-190µm/ 20-24	GCaMP6s hChr2-EYFP	1562 (syn)	ML: 948; AP: 784/ 4.6 Hz (5 planes)
sp4/Opto2p (selective)	VGATCHRCAMP2/B1-C1	~110-130-150-170-190µm / 26-30	GCaMP6s hChr2-EYFP	1595 (syn)	ML: 948; AP: 784/ 4.6 Hz (5 planes)
sp5/Opto2p(dual & selective)	VGATCHRCAMP4 / C1-D1	~110-130-150-170-190µm / 20-24	GCaMP6s hChr2-tdtomato	2459 (syn)	ML: 1185; AP: 980/ 4.6 Hz (5 planes)

Supplementary Table 1: Summary of 2p imaging FOVs.

[1] syn stands for hsyn1 human synapsin

[2]ML stands for medio-lateral axis, AP for antero-posterior

**Data**	**N points used for the test**	**Grouping variable**	**Statistical test**	**Mean/Effect size [CI 95%]**	**P Value**	**Note on trials inclusion**
Fig. 2E<br>Selectivity index in IN versus EN	267 INs vs 974 ENs	3 mice	LME<br>'SI~ Ntype +(1|animal)'	-0.053<br>[-0.11; 0.00]	0.051	Trial with single stim. discrimination (i.e. F1/F2 = 90/0 or 0/90)
Fig. 2G 'Princip. pop.' Testing 3 levels of PW stim. frequencies	18 Columns	5 mice	LME<br>'Resp.Princ.~ PW+(1| animal)'	0.66<br>[0.46; 0.87]	<0.001	Trials with RT > 0 sec; with the detailed PW/AW pairs (available in 9 FOV)
Fig. 2G 'Princip. pop.' Testing 3 levels of AW stim. frequencies	18 Columns	5 mice	LME<br>'Resp.Prin.~ AW+(1| animal)'	-0.18<br>[-0.44; 0.09]	0.21	as above
Fig. 2G 'Adj. pop.' Testing 3 levels of AW stim. frequencies	18 Columns	5 mice	LME<br>'Resp.Adj.~ PW+(1| animal)'	0.82<br>[0.67; 0.98]	<0.001	as above
Fig. 2G 'Adj. pop.' Response Testing 3 levels of PW stim. frequencies	18 Columns	5 mice	LME<br>'Resp.Adj.~ PW+(1| animal)'	0.33<br>[0.07; 0.59]	0.009	as above
Fig. 3I<br>(A) W1 blocking effect on Right choice<br><br>(B) W2 blocking effect on Right choice	15412 trials	8 mice	GLME<br>'Right choice~ F1 +F2+W1 block +W2 Block+(1| animal)'	(A) 0.09<br>[0.06; 0.13]<br><br>(B) -0.06<br>[-0.09; -0.03]	<0.001<br><br><0.001	Opto/sham paired trials with W1 or W2 blocking, all F1/F2 condition ; sessions with >65% correct (without opto)
Fig. 3G<br>W1&W2 blocking effect on Correct response	9298 trials	10 mice	GLME<br>'Correct choice~ |F1-F2| + F1+F2 + Block+(1| animal)'	-0.35<br>[-0.44; -0.27]	<0.001	Opto/sham paired trials with W1&W2 blocking; all F1/F2 condition; sessions with >65% correct (without opto)
Fig. 4B<br>$ß_{F1}$ vs $ß_{F2}$	3706 neurons	7 mice	LME<br>' $ß_{F1}$~ $ß_{F2}$+(1| animal)'	0.38<br>[0.31; 0.45]	<0.001	Trials with RT > 0 sec;
Fig. 4B<br>$ß_{F21}$ vs B_{F1xF2}	3706 neurons	7 mice	LME<br>' $ß_{F1}$~ B_{F1xF22}+(1| animal)'	-0.61<br>[-0.69; -0.53]	<0.001	as above
(not shown ~Fig. 4B)<br>B_{F2} vs B_{F1xF2}	3706 neurons	7 mice	LME<br>'B_{F2}~ B_{F1xF2}+(1| animal)'	-0.60<br>[-0.66; -0.53]	<0.001	as above
Fig. 5C<br>neurom. slope<br>(A) Rw1-Rw2 vs Rw1<br>(B) Rw1-Rw2 vs bhv<br>(C) Rw1 vs Rw2<br>(D) Rw1 vs bhv	11 FOV	7 mice	LME with post hoc comparison<br>slope~ item[3] +(1|animal)	(A) 0.71[1]<br>(B) 0.75<br>(C) 0.08<br>(D) 0.04	<0.001<br><0.001<br>0.69<br>0.85	Trials with RT > 0 sec; animal engaged; F1+F2 =90; sessions with >65% correct
Fig. 5C<br>neurom. lapse<br>(A) Rw1-Rw2 vs Rw1<br>(B) Rw1-Rw2 vs bhv<br>(C) Rw1 vs Rw2<br>(D) Rw1 vs bhv	11 FOV	7 mice	LME with post hoc comparison<br><br>lapse~ item[3] +(1|animal)	(A) -0.05[1]<br>(B) -0.20<br>(C) 0.004<br>(D) -0.15	0.005<br><0.001<br>0.81<br><0.001	Trials with RT > 0 sec; animal engaged; F1+F2 =90; sessions with >65% correct
Fig. 5D<br>Neurom. bias<br>Left vs Right choice	11 FOV	7 mice	LME<br>bias ~ item[3] +(1|animal)	(A) 3.1 [-2.6; 8.8]<br><br>(B) -1.7 [-12.8; 9.4]	0.26<br><br>0.75	Animal engaged; F1+F2 =90; sessions with >65% correct<br>(A) Trials with RT > 0 sec<br>(B) Trials with RT > 1 sec
Fig. 5D<br>Neurom. slope<br>Left vs Right choice	11 FOV	7 mice	LME<br>slope~ item[3] +(1|animal)	(A) -0.11 [-0.49; 0.27]<br><br>(B) -1.7 [-4.9; 1.5]	0.55<br><br>0.28	as above<br>(A) Trials with RT > 0 sec<br>(B) Trials with RT > 1 sec
Fig 5E<br>Neurom. bias<br>Engaged vs Disengaged	11 FOV	7 mice	LME<br>bias ~ item[3] +(1|animal)	(A) 4.0 [-2.8 ; 10.8]<br><br>(B) 2.9 [-6.0; 11.8]	0.24<br><br>0.51	Animal engaged; F1+F2 =90; sessions with >65% correct;<br>(A) Trials with RT > 0 sec<br>(B) Trials with RT > 1 sec; At least 50 trials per FOV.

Fig 5E<br>Neurom. slope<br>Engaged vs Disengaged	11 FOV	7 mice	LME<br>slope~ item[3]<br>+(1|animal)	(A) -0.43 [-0.94; 0.07]<br>(B) -1.5 [-3.9; 0.9]	0.088<br>0.200	same as above<br>(A) Trials with RT > 0 sec<br>(B) Trials with RT > 1 sec
Fig. 6A & B<br>change in AUROC<br>W1 vs W2 pop.<br>(A) AUROC target side<br>(B) AUROC choice side	(A) 9 FOV	5 mice	LME<br>AUROC~ item[3]<br>+(1|animal)	(A) 0.067 [0.047; 0.087]<br>(B) 0.004 [-0.014; 0.023]	<0.001<br>0.64	Trials with RT > 1sec; stim. condition paired for left and right choice; |F1-F2|<30Hz; sessions with >65% correct. At least 50 trials per FOV.
Fig. 6C<br>correlation b/w choice and sensory discrimination<br>(A) all neurons<br>(B) 20% "best sensory" neurons<br>(C) 20% "best choice" neurons<br>(D) 20% most sensory-choice interaction neurons [2]<br>(E) 20% most multiwhisker suppression neurons	(A) 3118 neurons<br>(B) 311 neurons<br>(C) 311 neurons<br>(D) 311 neurons<br>(E) 311 neurons	5 mice<br>(9 FOV)	LME<br>($ß_{C1}$- $ß_{C2}$)~ ($ß_{F1}$- $ß_{F2}$)<br>+(1|animal)	(A) -0.003 [-0.04; 0.03]<br>(B) 0.05 [-0.06; 0.16]<br>(C) 0.025 [-0.07; 0.12]<br>(D) 0.70 [0.63; 0.78]<br>(E) -0.09 [-0.21; 0.02]<br>0.62531 0.78368	0.88<br>0.37<br>0.62<br><0.001<br>0.09	Trials with RT > 1sec; stim. condition paired for left and right choice; |F1-F2|<30Hz; sessions with >65% correct; At least 50 trials per FOV.
Fig. S7<br>pop CPs (choice information)<br>(A) Rc1<br>(B) Rc2<br>(C) Rc1-Rc2	11 FOV	7 mice	LME<br>Cp~ (Rc) +(1|animal)	(A) 0.63 [0.60; 0.65]<br>(B) 0.59 [0.56; 0.62]<br>(C) 0.69 [0.66; 0.71]	<0.001<br><0.001<br><0.001	Trials with RT > 1sec; stim. condition paired for left and right choice; sessions with >65% correct; At least 50 trials per FOV.
Fig. S7<br>pop CPs multiple comparison (choice information)<br>(A) Rc1 vs Rc2<br>(B) Rc1 vs (Rc1-RC)<br>(C) Rc2 vs (Rc1-RC)	11 FOV	7 mice	LME with post hoc comparison<br>Cp~ (Rc) +(1|animal)	(A) 0.037 [1]<br>(B) -0.061<br>(C) -0.098	<0.008 (overlapping CI)<br><0.001<br><0.001	same as above
Fig. 7C<br>correlation b/w engagement modulation and sensory discrimination<br>all neurons	3706 neurons	7 mice	LME<br>Beng~ (BF1-BF2)<br>+(1|animal)	-0.03 [-0.06; 0.00]	0.08	Trials with RT > 1sec; stim. condition paired for engaged and disengaged trials
Fig. 7G<br>correlation b/w engagement weights and sensory weights<br>(A) $ß_{F1}$<br>(B) $ß_{F2}$<br>(C) $ß_{F1xF2}$	3706 neurons	7 mice)	LME<br>Beng~ $ß_{F1}$ + $ß_{F2}$ + $ß_{F1xF2}$ + (1|animal)	(A) -0.01 [-0.12; 0.10]<br>(B) -0.06 [-0.16; 0.04]<br>(C) -0.60 [-0.77; -0.42]	0.85<br>0.24<br>< 0.001	Trials with RT > 1sec; stim. condition paired for engaged and disengaged trials<br>NOTE: $ß_{F1xF2}$ alone capture the dependence
Fig. 7H<br>engagement related change in evoked firing rate<br>(A) Rw1<br>(B) Rw2<br>(C) Rw1xw2	11 FOV	7 mice	LME<br>Beng~ item<br>+(1|animal)	(A) 0.21 [0.11; 0.30]<br>(B) 0.24 [0.14; 0.33]<br>(C) 0.03 [-0.07; 0.12]	<0.001<br><0.001<br>0.55	Trials with RT > 1sec; stim. condition paired for engaged and disengaged trials
Fig. 7H<br>Multiple comparison<br>(A) Rw1 vs Rw2<br>(B) Rw1 vs Rw1xw2<br>(C) Rw2 vs Rw1xw2	11 FOV	7 mice	Engmod ~ 1 + Item + (1 | animal)	(A) -0.03 [1]<br>(B) 0.18<br>(C) 0.21	0.61<br>0.004 (overlapping CI)<br><0.001	Trials with RT > 1sec; stim. condition paired for engaged and disengaged trials

Supplementary Table 2; summary statistics: All tests are two-sided. Statistical tests are grouped figure wise and/or topically, when the test was repeated with variations, variations are indicated by letters (A), (B), … These use the same model but e.g., a different set of trials or a different population of neurons. [1]There is no confidence interval for post-hoc comparison using LME, but we noted when confidence intervals of effect estimate were overlapping. [2] "sensory-choice interaction" is computed as the absolute value $|(ß_{F1} - ß_{F2}) * (ß_{C1} - ß_{C2})|$, i.e. the product of correct sensory and choice discrimination weights. [3]"item" relates to a Boolean variable use to set the comparison in the first column: e.g. Rw1 vs Rw2 is coded as 0 and 1s.
