# Supplementary material for: Coexistence of state, choice, and sensory integration coding in barrel cortex LII/III
Source: Nat Commun. 2024 Jun 5;15:4782. doi: 10.1038/s41467-024-49129-9 (PMC11153558; doi:10.1038/s41467-024-49129-9)
Supplement: Supplementary file 3 — Description of Additional Supplementary Files [file 41467_2024_49129_MOESM3_ESM.pdf]

File name: Supplementary Movie 1
Description: Example video and tracking of whisker (black), nose (red) and tongue (magenta) movements. Dashed vertical line depicts detected reaction time. Whisker stimulus is provided between 1 and 2 s. Example trials were chosen to include trials with movements prior to stimulus onset, trials with movements during whisker stimulation and trials with withheld movement until end of the stimulus.
