# Supplementary material for: Coexistence of state, choice, and sensory integration coding in barrel cortex LII/III
Source: Nat Commun. 2024 Jun 5;15:4782. doi: 10.1038/s41467-024-49129-9 (PMC11153558; doi:10.1038/s41467-024-49129-9)
Supplement: Supplementary file 5 — Reporting Summary [file 41467_2024_49129_MOESM5_ESM.pdf]

nature portfolio

Corresponding author(s): Florent Haiss, Pierre-Marie Garderes

Last updated by author(s): Apr 16, 2024

Reporting Summary

Nature Portfolio wishes to improve the reproducibility of the work that we publish. This form provides structure for consistency and transparency in reporting. For further information on Nature Portfolio policies, see our Editorial Policies and the Editorial Policy Checklist.

Statistics

For all statistical analyses, confirm that the following items are present in the figure legend, table legend, main text, or Methods section.

n/a	Confirmed	
☐	☒	The exact sample size (*n*) for each experimental group/condition, given as a discrete number and unit of measurement
☐	☒	A statement on whether measurements were taken from distinct samples or whether the same sample was measured repeatedly
☐	☒	The statistical test(s) used AND whether they are one- or two-sided<br>*Only common tests should be described solely by name; describe more complex techniques in the Methods section.*
☒	☐	A description of all covariates tested
☒	☐	A description of any assumptions or corrections, such as tests of normality and adjustment for multiple comparisons
☐	☒	A full description of the statistical parameters including central tendency (e.g. means) or other basic estimates (e.g. regression coefficient) AND variation (e.g. standard deviation) or associated estimates of uncertainty (e.g. confidence intervals)
☐	☒	For null hypothesis testing, the test statistic (e.g. *F*, *t*, *r*) with confidence intervals, effect sizes, degrees of freedom and *P* value noted<br>*Give P values as exact values whenever suitable.*
☒	☐	For Bayesian analysis, information on the choice of priors and Markov chain Monte Carlo settings
☒	☐	For hierarchical and complex designs, identification of the appropriate level for tests and full reporting of outcomes
☐	☒	Estimates of effect sizes (e.g. Cohen's *d*, Pearson's *r*), indicating how they were calculated

Our web collection on statistics for biologists contains articles on many of the points above.

Software and code

Policy information about availability of computer code

Data collection	ScanImage 2018 (Vidrio technoloy); Custom LabVIEW code for behavioral data;
Data analysis	Suite 2p (MATLAB) for calcium imaging pre-processing; Custom MATLAB (2018a) code for further data analysis; ImageJ version 1.54f

For manuscripts utilizing custom algorithms or software that are central to the research but not yet described in published literature, software must be made available to editors and reviewers. We strongly encourage code deposition in a community repository (e.g. GitHub). See the Nature Portfolio guidelines for submitting code & software for further information.

Data

Policy information about availability of data

All manuscripts must include a data availability statement. This statement should provide the following information, where applicable:

- Accession codes, unique identifiers, or web links for publicly available datasets
- A description of any restrictions on data availability
- For clinical datasets or third party data, please ensure that the statement adheres to our policy

Data will be made available upon request

Research involving human participants, their data, or biological material

Policy information about studies with human participants or human data. See also policy information about sex, gender (identity/presentation), and sexual orientation and race, ethnicity and racism.

Reporting on sex and gender	N/A
Reporting on race, ethnicity, or other socially relevant groupings	N/A
Population characteristics	N/A
Recruitment	N/A
Ethics oversight	N/A

Note that full information on the approval of the study protocol must also be provided in the manuscript.

Field-specific reporting

Please select the one below that is the best fit for your research. If you are not sure, read the appropriate sections before making your selection.

☒ Life sciences ☐ Behavioural & social sciences ☐ Ecological, evolutionary & environmental sciences

For a reference copy of the document with all sections, see nature.com/documents/nr-reporting-summary-flat.pdf

Life sciences study design

All studies must disclose on these points even when the disclosure is negative.

Sample size	We planned 10 to 12 animals per experimental technique. With a power of 0.8 (1- Beta) and an alpha error probability of 0.05, 10 animals per experimental technique are sufficient to demonstrate a strong effect Cohen's D ~1, (without accounting for data exclusion described below).
Data exclusions	All animals with a healthy cranial window preparation were included. Exclusion was applied only if the cranial window preparation degraded before the animal reached expert performance in the behavioral task. (visible scar tissue preventing imaging/optogenetic for 3 and 1 animal respectively).
Replication	All replication information and data are presented in the manuscript.
Randomization	No experimental groups were used. statistical test were performed within subject between experimental condition.
Blinding	Blinding was not necessary as different experimental conditions were intersperesed and randomized by the behavioral algorithm (stimuli & optogenetic) or subject to specific analysis (behavioral outcome).

Reporting for specific materials, systems and methods

We require information from authors about some types of materials, experimental systems and methods used in many studies. Here, indicate whether each material, system or method listed is relevant to your study. If you are not sure if a list item applies to your research, read the appropriate section before selecting a response.

Materials & experimental systems

n/a	Involved in the study	
☒	☐	Antibodies
☒	☐	Eukaryotic cell lines
☒	☐	Palaeontology and archaeology
☐	☒	Animals and other organisms
☒	☐	Clinical data
☒	☐	Dual use research of concern
☒	☐	Plants

Methods

n/a	Involved in the study	
☒	☐	ChIP-seq
☒	☐	Flow cytometry
☒	☐	MRI-based neuroimaging

Animals and other research organisms

Policy information about studies involving animals; ARRIVE guidelines recommended for reporting animal research, and Sex and Gender in Research

Laboratory animals	7 C57BL/6J, 2 GAD-67-cre and 1 VGAT-cre male mice for imaging experiments; 2 GAD-67-cre and 9 VGAT-cre male mice for optogenetic experiments.
Wild animals	No wild animals were used in the study.
Reporting on sex	Only male mice were used for the experiment. A single sex was chosen to minimize variability of the measurments.
Field-collected samples	No field collected samples were used in the study.
Ethics oversight	All experiments were conducted in accordance with the guidelines on the ethical use of animals from the European Community Council Directive of 24 November 1986 (86/609/EEC) and in accordance with institutional animal welfare guidelines and were approved by Animalerie Centrale, Médecine du Travail and the Ethics Committee CETEA of Institut Pasteur (protocol number : dap190164).

Note that full information on the approval of the study protocol must also be provided in the manuscript.
